# Nano-Magnetic Resonance Imaging (Nano-MRI) Gives Personalized Medicine a New Perspective

**DOI:** 10.3390/biomedicines5010007

**Published:** 2017-02-01

**Authors:** Lorenzo Rosa, Jonathan Blackledge, Albert Boretti

**Affiliations:** 1 Centre for Microphotonics (H74), Swinburne University of Technology, Hawthorn 3122, Australia; lrosa@ieee.org; 2 Department of Information Engineering, University of Parma, Parma 43121, Italy; 3 Military Technological College, Muscat 111, Oman; Jonathan.Blackledge@mtc.edu.om; 4 School of Mathematics, Statistics and Computer Science, University of KwaZulu-Natal, Durban 4000, South Africa; 5 School of Electrical and Electronic Engineering, Dublin Institute of Technology, Kevin Street, Dublin 8, Ireland; 6 Department of Computer Science, Faculty of Natural Science, University of Western Cape, Bellvile 7535, South Africa; 7 Department of Mechanical and Aerospace Engineering (MAE), Benjamin M. Statler College of Engineering and Mineral Resources, West Virginia University (WVU), Morgantown, WV 26506, USA

**Keywords:** personalized medicine, nano-MRI, recent developments

## Abstract

This paper reviews some of the major and most recent advances in nanoscale-magnetic resonance imaging (nano-MRI) for personalized medicine (PM). Nano-MRI may drastically expand the capabilities of the traditional magnetic resonance images (MRI), down to the nanometer scale and possibly, in the near future, at the atomic scale. Nano-MRI is potentially able to observe structures which cannot be seen using today’s molecular imaging, with sensitivities of many billions of times better than MRI as currently used in hospitals, for example. The paper briefly reports on the foremost research themes in nano-MRI.

## 1. Introduction

This work provides a brief overview of molecular imaging techniques and its present and future potential in personalized medicine, with special a focus on the magnetic resonance imaging (MRI) approach. It discusses the current techniques that allow for the in vivo visualization of molecular processes at the nanoscale resolution (nano-MRI).

Nano-MRI is progressing rapidly thanks to the work of a very small but extremely brilliant community of experts. This paper is not intended to be a comprehensive review of nano-MRI written for these experts, but rather a concise description of the present achievements for a much broader audience of medical professionals. The goal is to bridge the gap between the nano-MRI community and those in the medical field that will ultimately benefit from the further development of nano-MRI targeting specific medical goals. The aim of this review is to highlight the potential of nano-MRI in the improvement of MRI sensitivity and consequently on the impact of this widely-used technique for diagnosis and personalized treatment. Sensitivity improvements are based on the use of magnetic nano probes in conventional MRI as well as novel nanoscale imaging based on nitrogen-vacancy (NV) centers in diamonds.

## 2. Personalized Medicine (PM)

Early diagnosis and treatments tailored precisely to an individual patient are the primary goals of personalized medicine (PM), conceived as a system for delivery of the necessary treatment with the best timing for a specific patient. The inherent personalization in medical imaging is the ability for it to assess the geometry and location of abnormalities. This is a fundamental endeavor, the imaging of suitable biomarkers being instrumental in identifying candidates for early intervention. The ability to detect diseases before they show symptoms as well as the capacity to accurately deliver drugs, makes the principle of molecular imaging a highly sought after development. It can provide for the visualization of live tissue physiological, biochemical and biological processes, thereby permitting patient-specific tailoring of therapy, follow-up, and monitoring. It also enables the reduction of invasive treatments and opens the way to theranostics (therapeutics and diagnostics) [1].

Theranostics provide a link to nanotechnology as nanoparticle production techniques and materials are constantly improving, thereby making them useful both as probes for imaging and for carrying drugs into the body. This has the significant advantage of being able to deliver a high quantity of substance in relationship to its size. Agents currently studied range from formulas based on liposomal vesicles functionalized with cytostatic compounds and contrast media for magnetic resonance imaging (MRI), to carbon nanotubes used to carry radioisotopes for therapeutic and imaging purposes. This includes the use of dendriworms engineered to deliver small interfering ribonucleic acid (siRNA) for MRI or optical visualization. A new strategy in this field involves using cells for both diagnostics and therapy, an approach dubbed “cellular theranostics” [1].

PM is a new medical paradigm aiming to anticipate a diagnosis and provide treatments with individual specifics, for delivery of the necessary treatment with the best timing to a specific patient. A fundamental aspect of PM is medical imaging, for purposes such as differential diagnosis, adaptation to the specific patient features, disease screening and preventative intervention based on the response of imaging biomarkers. Decisions about treatment(s) can then benefit from being able to visualize abnormalities in live tissue and detect process alterations of the biochemical and physiological kind, making use of structural visualization and molecular imaging. The drive for accurate biopsies to collect biological specimens, detecting genetic morphologies by their linked biomarkers through radio genomics is also possible. Imaging also plays a fundamental role in planning patient-tailored therapy, monitoring, and follow-up, as well as establishing targets to make treatments in a non-invasive way, especially in a theranostic framework. The changes in training, clinical practice and in research brought by this new technique may bring significant changes in the training and preparation required of future professionals and the medical imaging fraternity in general.

## 3. Molecular Imaging (MI)

One key technique to implementing PM is molecular imaging. This provides the ability to image tissues in vivo without having to extract them from the organism for analysis. Early detection through cell and molecule visualization to monitor biological processes and local biochemistry are two applications of molecular imaging. In addition to disease staging, which is the determination of how severe a disease is, molecular imaging allows for tailored therapy and prognosis. For this purpose, it is necessary to be able to cover the whole extent of the body in a reasonable time with a high sensitivity to disease markers. The options in this respect are combined positron emission tomography (PET), computed tomography (CT) and diffusion-weighted MRI, which permits the disease to be staged and tailored for a treatment based on the results of a single examination [1].

A therapeutic agent can be delivered precisely to the site of the disease by using image guidance, regardless of the agents being chemicals such drugs and radio-agents, genetic materials, mechanical equipment such as stents, cellular material, acoustic perturbations as used for high-intensity focused ultrasound (HIFU), or temperature waves to produce hyperthermia or hypothermia. The image-guided procedure is specifically matched with local anatomy and functionality and the patient characteristics, becoming an integral part of PM. The effect can be triggered in a local environment and be timely, according to the therapeutic plan of the specific patient. This includes applications directed by non-invasive techniques such as MRI-guided HIFU, which is the only technology able to provoke a deep-tissue non-invasive temperature increase that is compatible with a clinical environment. The spatial-temporal tuning of the approach can be achieved by using MRI to map, in continuous way, the temperature effect of HIFU and predict the lesion formation as a function of the delivered heat dose [1].

Clinical imaging assists in the assessment of diseases by creating virtual images of the interior structures of a patient’s body, working in combination with pathology to achieve a correct diagnosis [2]. Molecular imaging has brought a fundamental change to clinical imaging, bringing imaging technology to molecular biology so that biochemical reactions in living organisms can be visualized at molecular scale in a non-intrusive way. One main feature of molecular imaging is the use of imaging probes, which are biomarkers that bind to the molecules to be imaged to permit their visualization. Applications for this technology promise to foster new therapeutic approaches for oncologic, neurological and cardiovascular diseases. In this context, PM will benefit from the ability of molecular imaging to allow for early diagnosis and the prediction of response to treatment, as well as the development of new drugs through the optimization of drug candidate tests in preclinical and clinical environments. Three emergent imaging techniques that are poised to give a large contribution to PM have specific imaging applications in apoptosis, angiogenesis, and receptors.

The recent bout of innovations in the field of biotechnology is leading to the emergence of the new discipline of molecular imaging. It is able to show in real time and in live tissues, the biochemical processes that sustain the process of a disease. It has been defined as “noninvasive imaging and quantification of molecular and biochemical events that occur at the cellular and molecular level of tissues in their normal surroundings inside living bodies” [3].

Biomarker probes attached to proteins involved in disease chemistry have a critical role in allowing molecular imaging within live cells without disturbing their function. This provides the ability to track in real time the spatial-temporal distribution of proteins with high target specificity. Designing and synthesizing molecular probes is thus the first and most important step in molecular imaging, where one has to design both the targeting and the signaling component. The targeting component has evolved from being a small molecule at the start of the field to fully-fledged peptides, proteins, antibodies, aptamers, affibodies, and nanoparticles, which contain the ligand to the target molecule or are surface-functionalized with it. The signaling component is conjugated or labeled to the targeting signal in a way that permits out-of-body detection by radioactive, magnetic, echogenic, luminescent or fluorescent means [2].

## 4. Nano-Magnetic Resonance Imaging (Nano-MRI)

Magnetic resonance (MR) is an imaging technique that relies on the electromagnetic response of tissues to a combination of magnetic and radio energy in order to allow for tissue imaging with an enhanced resolution in both space and time. The tissue contrast of MRI images is excellent and can penetrate tissues without depth limits, simultaneously acquiring information on anatomy and the functionality of structures. The weakness of the magnetic signal and the low detector sensitivity are usually overcome by introducing contrast molecules and amplification. The choice between ferromagnetic and paramagnetic agents determines the positive or negative contrast that can be obtained in the imaging process, reaching a resolution of a few number of cells. A major limitation of the clinical techniques being actively researched is the management of possible toxicity caused by the large dose of probes that are sometimes necessary for accurate imaging [2].

Nanoscale-magnetic resonance imaging (nano-MRI) is a new technique that promises to bring the resolution of MRI measurements, normally limited to tens of micrometers, down to the nanometric scale, making measurements of single-biomolecule MR spectra a tangible aspiration. Nano-MRI research includes techniques such as magnetic resonance force microscopy (MRFM), optically-detected magnetic resonance (ODMR) using nitrogen-vacancy (NV) centers and many other applications of quantum mechanics and nanotechnologies.

Diamond impurities on the atomic scale have been used as spin sensors to obtain remarkable results in nano-MRI, by exploiting interactions between magnetic dipoles in place of detection by induction means. The most important nano-MRI results in single-biomolecule imaging in vivo on the nanometric scale at room-temperature conditions. This has been obtained by utilizing NV centers in diamonds as sensors [4].

Nuclear spins are characterized by the spin-lattice relaxation time, whose limited length affects the use of hyperpolarized media and weak spin-spin interactions in nano-MRI, and can be extended by using nuclear spin singlet states. Due to the difference in symmetry, there is no interaction between associated singlet and triplet states, and, the only way for relaxation of the singlet state is by weak processes of higher order. This involves adjacent spins, with the same difference bars being obtained from accessing the singlet states by using radio frequency (RF) pulses. To address this, specific sequences of pulses such as M2S and spin-lock induced crossing (SLIC) have been designed [5] in order to transfer the triplet state polarization by exploiting the coupling peculiarities of nearly equivalent spins with regard to their spectroscopic features, in particular, the J-coupling parameter and the differences in their chemical shift.

Experiments [6] to date have shown that it is possible to measure differences in J-coupling down to 10 mHz, enough to determine the structure of protein molecules, as the difference between the syn and anti-geometries is reflected in the J-couplings. Long-range detection of such coupling differences is limited by the resolution of couplings, which is around 100 mHz, since the lifetime of protonic spins is limited at a scale of seconds, though the transfer to the singlet state can overcome this limit by extending the technique to weaker long-range couplings up to five or more bonds away.

Magnetic nano-probes [7] have a strong potential for improving the performance of cancer-related MRI, as contrast agents for MRI relying on magnetic nanoparticles can act as probes for imaging specific tumors. Significant improvements in the synthesis and functionalization of their surface with biocompatible chemicals have recently been affected, leading to multimodal imaging. Both engineered and natural coverings of the surface such as coronas of proteins are important in making MRI nano-probes biocompatible and improving their performance. However, this burgeoning field still has to cope with several challenges in order to progress further.

MR force microscopy [8] has been used to image the chemical shift of solids with 1-µm accuracy for a single bi-dimensional spectrum. This provides the possibility of a 3D extension with multiple spectral lines. The measurement time is reduced by multiplexing in frequency and space by either a Fourier or a Hadamard transform method. The space dimension is acquired by quadrature detection combined with Hadamard encoding.

Medical diagnosis greatly benefits from imaging technologies such as MRI by the ability to visualize in vivo internal tissues, and, the activity of the brain through functional MRI (fMRI), with a resolution on the millimeter scale or less [9]. While this is enough for imaging body tissue structures such as organs, it cannot readily visualize molecules, for which the resolution required is in the scale of a nanometer or less. This is the realm in which nano-MRI is being developed with a variety of techniques to support molecular biology in achieving single-molecule resolution.

## 5. Nitrogen-Vacancy (NV) Center Magnetometry

The understanding of the physical-chemical processes of magnetism and the latest spectacular developments in magnetic data storage are both underpinned by magnetic imaging. This involves overcoming the limits associated with standard approaches such as the magneto-optic Kerr effect for X-ray and electron microscopy with respect to high-resolution real-time imaging [10]. NV center spins in diamonds can be used to build an array of magneto-optical sensors to image the stray magnetic field patterns from thin ferromagnetic films with sub-micrometric accuracies of over a 100 × 100 µm^2^ surface at frame rates compatible with video recording. This technique makes it possible to image magnetic structures without applying a microwave signal by exploiting all-optical spin relaxation contrast imaging, which can be extended to sensitivities below µT and spatio-temporal resolutions below light wavelength and millisecond scales, respectively. This practical method for wide-field microscopy can apply to phenomena such as domain wall and skyrmion dynamics and the spin Hall effect in metals.

Organic and biological molecules can be analyzed using nuclear magnetic resonance (NMR) spectroscopy only when a sufficiently large sample is available [11]. The combination of two quantum bits, which creates a system akin to the coupling of an electronic spin with an ancillary nuclear spin, can be used to sense the MR spectroscopy at room temperature of single ubiquitin proteins with several nuclear species when they are linked to the surface of a diamond containing qubits. By exploiting the quantum logic relationships between qubits to improve readout faithfulness and a surface treatment to improve the NV center spin coherence, sensitivities can be pushed to the level where single proton spins are detectable with an integration window of only 1 s. This permits users to rapidly and reliably recognize single proteins and derive their chemical composition by spectral methods.

The creation of singlet states in molecules is a process requiring significant microwave radio frequency (RF) power. This limits its practical use because of safety concerns for in vivo biological materials. It was recently experimentally and theoretically demonstrated that RF spin-locking fields two orders of magnitude weaker than conventional RF methods, and thus safer for biomaterials, could also create long-lifetime nuclear spin singlet states, with a long singlet-triplet coherence time that can be maintained after the spin-locking field is removed [12].

Long lived nuclear singlet states have been proposed as a new contrast mechanism in NMR and MRI. The new NMR contrast mechanism named SUCCESS (Suppression of Undesired Chemicals using Contrast-Enhancing Singlet States) has recently been demonstrated in vitro, in order to improve the ability to identify unresolved or hidden peaks buried in the background. It utilizes a quantum filter based on nuclear spin singlet states created in the target molecule, preserved by a continuous RF field, and then converted back into a detectable state after saturating the magnetization of extraneous molecules, by a tailored pulse sequence [13].

The ability to image bio-magnetic structures inside cells with a high resolution of 400 nm has been demonstrated in magnetotactic bacteria in ambient conditions by placing them on a nanometric layer array of NV centers on a diamond surface. The quantum spin states are optically probed and allow for rapid and faithful imaging of the chains of magnetosome particles inside the bacteria, and to correlate the magnetic field with the simultaneously captured optical image. Submicron resolution on a 100-micron field of view has been achieved, permitting the location and measurement single magnetosomes [14].

Bulk-matter NMR spectroscopy and MRI of multiple nuclear species has been achieved in structured samples by using single shallow NV centers in diamonds, permitting the imaging of nanometric proton spin ensembles in ambient conditions with moderate fields of 20 mT, two orders of magnitude less than conventional MRI [15].

A new experimental method has been devised for measuring the depth of NV centers in diamond with accuracy of around 1 nm. Single NV centers are imaged simultaneously by confocal microscopy and NMR and their interaction with proton spins is modeled through polarization statistics, achieving reasonable agreement with ion implantation simulations [16].

**Figure 1:**
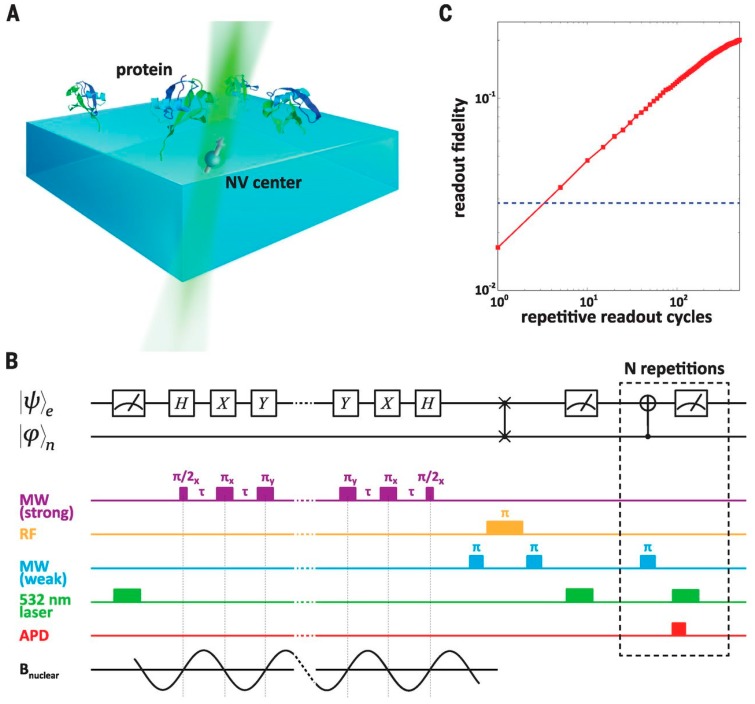
Experimental setup and magnetometry with repetitive readout described in [11]. Image from [11]. Reprinted with permission from AAAS. (**A**) Schematic of the experimental setup. Ubiquitin proteins attached to the diamond surface are probed using a proximal quantum sensor consisting of a nitrogen-vacancy (NV) center electronic spin and its associated 15 N nuclear spin; (**B**) Quantum circuit diagram and experimental magnetometry pulse sequence; (**C**) Measured gain in the readout fidelity as a function of repetitive readout cycles. MW: microwave; RF: radiofrequency; APD: avalanche photodiode; B magnetic field of the nuclear spin.

Figure 1 presents the experimental set-up of the magnetometry with repetitive readout as described in [11]. This work shows the opportunity to achieve nuclear magnetic resonance detection and spectroscopy of single proteins. Individual Fourier components are measured of the time-varying magnetic field created by a statistically polarized subset of proximal nuclear spins contained within a protein. The transverse magnetization of the spin ensemble undergoes precession at the nuclear Larmor frequency with a phase and amplitude that vary stochastically with every repetition of the sequence. Averaging over many iterations yields a zero mean magnetization but a nonzero variance, which results in a measurable magnetic resonance signal. To use the NV center as a sensor, its spin state is manipulated with a series of periodic microwave pulses separated by free-evolution intervals of length τ. This periodic modulation of the NV center spin creates a narrow band-pass frequency filter, allowing phase accumulation when the modulation frequency, defined as 1/τ, is close to twice the nuclear Larmor frequency. Varying the spacing between the π pulses yields a frequency spectrum that encodes information about the nuclear spins within the protein. Various other set-ups have been proposed based on NV centers in diamond sensors to achieve extremely high resolutions in both space and time.

Neuron in vivo imaging involves the measurement of the tiny magnetic fields produced by the action potentials, something that can be achieved by NV center-based magnetometry. To understand the dynamics of a neuron complex, it is necessary to image at a sub-cellular or synapse scale spatial resolution, aiming to image the dynamics of a single neuron inside the whole living organism. The challenge of applying NV-based magnetometry is the typical neuronal pulse duration of 2 ms, with a peak neural magnetic field value ≤ 10 nT at 100 nm of an axon surface. A model has been developed based on mimicking the temporal dynamics of an axon including an ensemble of NV centers contained in a single crystal ultrapure diamond membrane substrate. For detection, wide-field NV photoluminescence imaging was combined with confocal microscopy. The magnetic field is detected by either continuous ODMR or free induction decay, the two methods providing a similar sensitivity of 10 μT·Hz^−1/2^. To permit a sensitivity down to a single axon, the method needs to be applied within a specific sequence repetition and sensing volume (1 μm^3^) depending on the axon size. Currently, NV-magnetometry can achieve a single neuron scale (the whole organism scale) with no labelling at a 10-nm spatial resolution, 30-μs temporal resolution and 1-mm field of view. The method is non-invasive and non-toxic, allowing observation for an extended period without adverse effects on the live animal. This has been demonstrated by a single crystal diamond chip, used with a uniform 13-μm layer of high density NV (3 × 10^7^ cm^−3^) on the surface, where the biological sample is placed. The action potential magnetic field is detected as a time variance of the center of the NV magnetic resonance frequency with a temporal resolution of 32 µs and a magnetic field sensitivity of 15 pT/Hz^1/2^. The magnetic field measurement for an excised single neuron, peak to peak, is ~4 nT.

The biological processes of interest for biomedical imaging have very different scales, ranging from microns to nanometers, which requires nano-MRI for an understanding of them at the molecular scale in order to evaluate their biological functionality down to assessing the structure and electron configuration of single proteins and DNA sequences and imaging their engineering dynamics. To date, the technique has relied on powerful magnetic pulses, but now it is necessary to shift to greater sensitivity of detection and hyperpolarization methods. MRI at the molecular level looks forward to availing itself of sensors the size of a single atomic spin by using novel optical probes [4].

Diamond NV centers are employed in nano-MRI as sensing spins experiencing magnetic dipole-dipole interactions with other spins in close proximity, thus dispensing with the high magnetic fields of standard MRI and allowing for resolutions in the order of a nanometer with high wide-band SNR (signal-to-noise-ratio). Optical confocal and wide-field microscopies have been proposed as the foundation for NMR measurements and imaging of small groups of nuclear spins in polymers. This generates MW (microwave) pulse sequences of greater complexity to reduce the sensitivity to frequency of the coherence time of NV electronic spins, and, functionalizing scanning magnetic probes with nuclear spins in order to detect 2D proton images with nanometer-scale resolution and 3D images of dark electronic spins with even smaller resolution [4].

The NV center characteristics of photostable fluorescence and atomic size allow for its use as NMR sensors in nanodiamonds of sizes smaller than 10 nm. Its electron spin can be polarized optically and detected by observing its luminescence while issuing a sequence of MW pulses. In particular, Hanh-Echo MW pulse sequences permit the measurement of dephasing on the order of milliseconds in ultrapure bulk diamond grown isotopically with room-temperature processes, and dynamic decoupling pulse sequences permitted to reach a coherence time of around 1 s at 77 K temperature. A dark-state spin-dependent transition is exploited to optically pump the excited triplet state of the NV center, permitting engagement of a spin-orbit effect coupling by applying a suitable MW signal to trigger a transition. This allows for the modulation of the fluorescence with an amplitude proportional to the magnetic field, while allowing for positional measurements with a precision on the order of the size of the NV center itself. Bulk ultrapure diamond NV centers have reached sensitivities around 40 nT/Hz^1/2^ for DC (direct current) magnetometry and around 10 nT/Hz^1/2^ for AC (alternating current) magnetometry. NV center-based sensors have a weak interaction with nuclear spins, limiting the possibilities of MRI of single nuclear spins. However, a single NV center can achieve a 2D resolution of 12 nm for imaging of 1H NMR in polymers [4].

To achieve nanometric resolution in the localization of an NV center with purely optical methods, super-resolution methods based on stimulated depletion emission microscopy (STED) and stochastic optical reconstruction microscopy (STORM) must be employed. Therefore alternative methods combining STED and STORM with spin resonance techniques (spin echo sequences) have to be implemented. One of these methods, called Fourier magnetic imaging (FMI), uses the Fourier (or k-space) phase-encoding of the NV electronic spins in a diamond sensor and has been applied to magnetic field sensing. FMI acquisition and processing methods applied to NV in diamond achieves imaging in the k-vector space providing a 3.5 nm resolution. Pulsed magnetic field gradients are specifically used to phase-encode the spatial information on NV electronic spins in the “k-space”. After measuring the wavenumber, an FFT (fast Fourier transform) transformation is applied in order to retrieve the image in the real space with a wide field of view and significant computing time reduction. This is advantageous with respect to real space imaging since it allows for spatial multiplexing with increased SNR and high acquisition rate; the sensing is compressed in wavenumber space and results in the simultaneous acquisition of all the NV centers within the field of view [17,18].

A straight expansion of the FMI and of the other techniques described here, in regard to further engineering, would be to consider the use of another solid-state quantum spin system based on defects in silicon carbide (SiC). SiC is a complementary metal–oxide–semiconductor (CMOS); a compatible material having an advanced manufacturability that could make the engineering of a probe much easier. The defects of SiC possess electronic states whose sharpness in spin and optical transitions make them more and more relevant for nanoscale sensing. SiC is also a much more widely-used material in manufacturing applications than diamonds, and, as such, benefits from more established fabrication techniques. Research has discovered six distinct defect types in the 4H poly-type of SiC (4H-SiC) with electronic bound states working as quantum bits similar to the NV centers. In particular, the single silicon-vacancy defect in SiC is a good candidate for nanoscale MRI, due to the capability for electrical excitations similar to other single defects in SiC, allowing the engineering of more compact probes. Furthermore, DC SiC magnetometry has been proved to be feasible without recurring to the radiofrequency excitation, by pure optical excitation obtaining an actual sensitivity of 87 nT/Hz^1/2^ and a projected sensitivity of 100 fT/Hz^1/2^, thus making it comparable with the NV center in diamonds [17].

## 6. Nanocrystals

Another technique with high ability to revolutionize the therapeutic and diagnostic paradigms of medicine is based on nanocrystals, though success has been limited to date due to the relatively recent deployment of the technique. Among the issues to be resolved is the improvement of biocompatibility and colloidal stability against physiological conditions, while preserving the wanted physical-chemical properties such as a small enough size. Once these issues are addressed, novel nano-bio hybrid systems can be developed with manifold abilities in the therapeutic realm and with ultra-high detection sensitivities [19]. The use of magnetic nanocrystals as vectors for drug delivery and probes for imaging and detection, in particular for MRI, has the potential to bring about a dramatic enhancement in biomedical imaging which necessitates compatibility with the biological environment [19]. The non-invasive and tomographic abilities with multiple dimensions of current MRI techniques position them as powerful tools for medical diagnosis. However these same techniques have issues with respect to sensitivity and time resolution but can be effectively addressed by introducing magnetic nanocrystals. By applying an external magnetic field to such nanocrystals surrounded by wet tissue, the water molecules (in particular, the protons) will be affected in that their processes of relaxation of the nuclear spins are perturbed by the magnetic spins induced in the nanocrystals. As this will shorten the spin-spin relaxation time T2 (the spin–spin relaxation is the mechanism by which the transverse component of the magnetization vector exponentially decays towards its equilibrium value in NMR and MRI, T2 is the time constant characterizing the signal decay), the MRI images will be darker. The result of the conjugation of nanocrystals with active biomaterials such as antibodies is to obtain both an effect in MR contrast and a selective identification of the target biomolecules, acting as agents for molecular imaging to signal events of genetic and chemical modifications in the tissue.

The many uses of nanocomposites to obtain an accurate diagnosis of diseases, and to provide effective therapy, is discussed in [20]. A theranostic nanoparticle [21] is divided into at least three components, i.e., biomedical payload, carrier, and surface modifier. Nanocomposites are formulated by introducing therapeutic agents such as drugs or genes and imaging agents such as magnetic nanocrystals. This enables diagnosis and therapy, for example, of cancers while monitoring drug behavior. The protocol of [20] is based on the synthesis of four pyrenyl-based polymers: pyrenyl polyethylene glycol (Py-PEG), pyrenyl dextran (Py-DEX), pyrenyl hyaluronan (Py-HA) and pyrenyl-conjugated hetero-functional PEG (pyrenyl PEG). Their use to produce multifunctional nanocomposites for applications including multimodal imaging, targeted cancer detection and pH-sensitive drug delivery is then considered.

The production of inorganic nanocrystals is surveyed in [22]. In MRI, nanocrystals produce contrasts themselves, iron oxides being the most explored in this regard. Nanocrystals may also be given a coating that generates MR contrast, gold nanoparticles coated with gadolinium chelates being an example. MR-active nanocrystals can be used for imaging of the vasculature, liver and other organs, as well as molecular imaging, cell tracking and theranostics.

**Figure 2:**
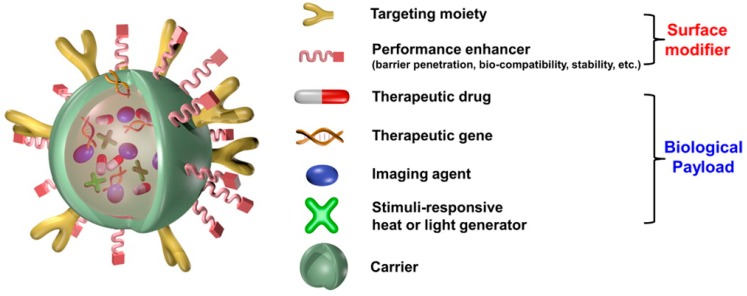
Schematic illustration of a multifunctional nanocomposite. Image reprinted with permission from [23]. Copyright (2015) American Chemical Society.

Figure 2 presents a schematic illustration of a multifunctional nanocomposite.

Authors of [24] present a scalable chemical vapor deposition method to synthesize FeCo/single-graphitic-shell nanocrystals that are soluble and stable in water solutions. The nanocrystals exhibit ultra-high saturation magnetization, r1 and r2 relaxations and high optical absorbance in the near-infrared region. Mesenchymal stem cells are able to internalize these nanoparticles, showing high negative-contrast enhancement in magnetic-resonance imaging (MRI).

Authors of [25] used semiconductor quantum dots and super-paramagnetic iron oxide nanocrystals for biomedical imaging. Quantum dots and super-paramagnetic iron oxide nanocrystals are embedded in the core of lipoproteins of the blood and it is demonstrated that the potential exists to image the kinetics of lipoprotein metabolism in vivo using fluorescence and dynamic MRI.

The magnetic properties of nanocrystals useful for this kind of imaging must give a high performance coupled with the possibility of easily traversing the mononuclear phagocytic system (MPS) for reliable expulsion from the biological system and the ability for active functionalization to achieve reliable linking to biomolecules. The protocols for synthesis of such conjugates by wet chemistry in water have general issues in delivering particles that are controlled in size, monodisperse, and with precise stoichiometry. These issues can be addressed by employing methods relying on non-hydrolytic injection at high temperature, which has been shown to produce useful contrast agents for MRI.

By producing nanocrystals with a size of 4, 6, 9, and 12 nm and subjecting them to a field of 1.5 T, higher contrasts are obtained for MRI at mass magnetization values of 25, 43, 80, and 102 emu/(g Fe), respectively [26]. The 9-nm nanocrystals are selected as a model probe for diagnosis of cancer and functionalized with a shell made of a conjugated ligand for Herceptin, a cancer-specific antibody, which binds to the receptor tyrosine-protein kinase erbB-2 protein HER2/neu receptor characteristic of cells in breast cancers (as demonstrated by binding to the breast cancer cell line SK-BR-3 ) which is known to over-express the HER2/neu marker. To prove the selective binding of the probes, images are obtained by T2-weighted MRI, which shows a significantly darker image with respect to the control cell line.

Dynamic 3D imaging of magnetic MRI contrast agents at clinical concentrations has been demonstrated [27] by magnetic particle imaging (MPI), a tomographic technique capable of real-time in vivo imaging with time resolution of 21.5 ms of a beating mouse heart while still being able to accurately resolve all the heart chambers.

Microspheres including gold and magnetic particles have been developed [28] for combined drug delivery and multimodal MR/CT imaging. The 25-um sized spheres are delivered by catheterism to treat malignant hepatic cancers and permit the visualization for targeted delivery via strong MRI T2 and CT contrast, confirming the selective drug administration.

**Figure 3:**
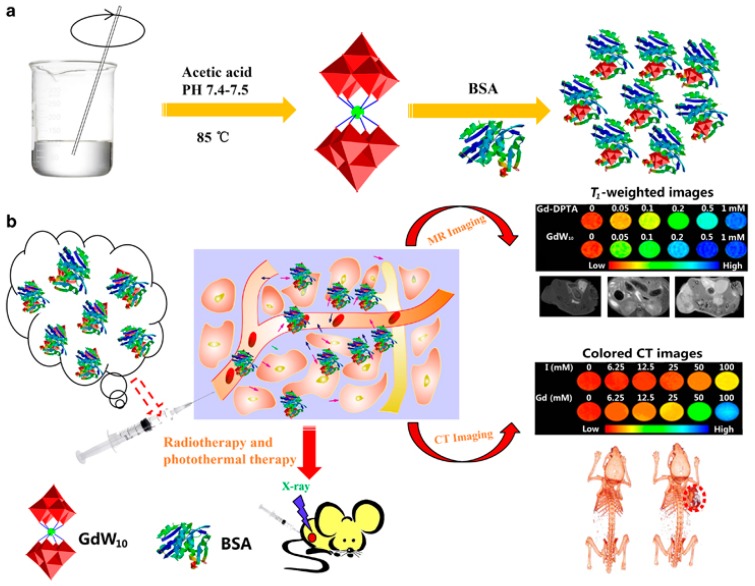
Schematic illustration of gadolinium polytungstates nanoclusters GdW10@BSA NCs for dual-modal magnetic resonance (MR) / computed tomography (CT) imaging-guided photothermal therapy/radiotherapy of cancer. (**a**) The synthesis process of GdW10@BSA; (**b**) The application of the as-made GdW10@BSA for bio-imaging and treatment of tumor. Image from [29]. Article distributed under a Creative Commons CC-BY license.

Gadolinium polytungstate nanoclusters have been synthesized [29] for the purpose of combining the enhancement of dual-modal MR/CT imaging with cancer theranostics properties on the nano-scale while avoiding side effects. The clusters show the ability to combine therapeutic enhancement of photothermal tumor ablation and radiotherapy with a low toxicity and an efficient clearance through renal function. Figure 3 presents a schematic illustration of gadolinium polytungstates nanoclusters GdW10@BSA NCs for dual-modal magnetic resonance (MR) / computed tomography (CT) imaging-guided photothermal therapy/radiotherapy of cancer.

Enhanced dual-mode CT/MR imaging of hepatocellular carcinoma with high X-ray attenuation intensity and relaxation has been shown in [30] by multifunctional gold nanoparticles, synthesized via dendrimer technology, that bind to the CD44—a cell-surface glycoprotein—receptor expressed by the cancer cells, combined with good dispersibility in water, stability, and cytocompatibility.

A new MRI contrast agent has been developed in [31], based on pathophysiological pH-sensitive response of CaP nanoparticles engineered to release Mn ions in the cancerous environment, which then bind to proteins and increase the contrast of the specific hypoxic regions, aiming for early detection of millimetric liver metastases.

**Figure 4:**
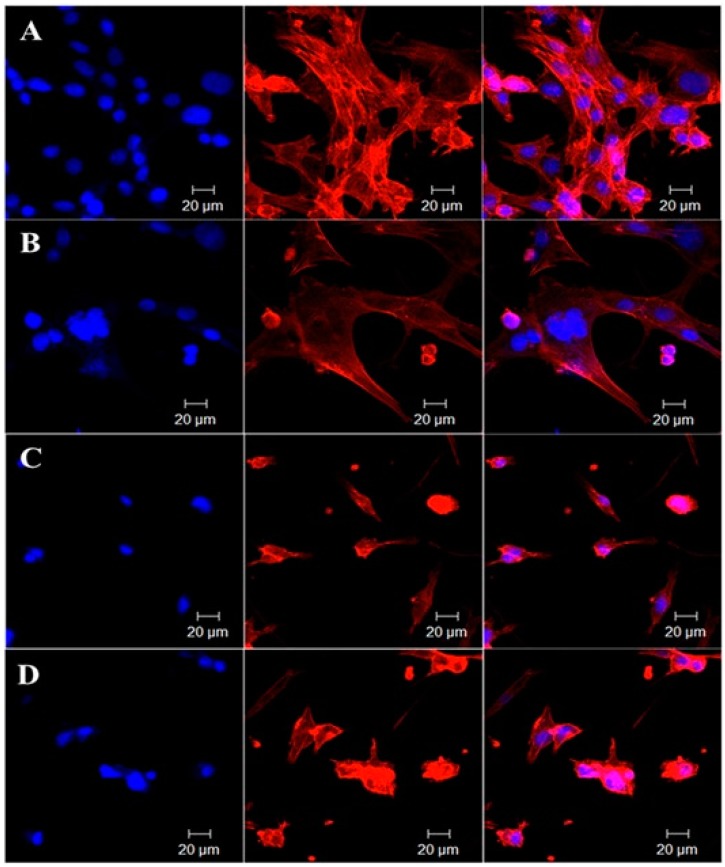
In vitro cytoskeletal imaging with rhodamine B displaying localized tumoricidal effects of (**A**) GO-IO (control); (**B**) GO-IO-MH; (**C**) GO-IO-DOX; and (**D**) GO-IO-DOX-MH. Image from [32]. Article distributed under a Creative Commons CC-BY license.

A new multifunctional graphene-based nanocomposite for cancer theranostic has been developed in [32] to combine magnetic field sensitivity for hyperthermic therapy and chemotherapy through pH-sensitive anticancer drug release, while offering enhanced performance as agent for T2-contrast MRI, as evidence by in vitro tests. Figure 4 presents the in vitro cytoskeletal imaging with Rhodamine B displaying localized tumoricidal effects of (A) GO-IO (control), (B) GO-IO-MH, (C) GO-IO-DOX, and (D) GO-IO-DOX-MH. (GO-IO Graphene Oxide–Iron Oxide; GO-IO-DOX: Graphene Oxide–Iron Oxide-Doxorubicin nanocomposite).

## 7. Conclusions

We have provided a brief overview of nano-MRI applications in personalized medicine. The paper is a concise description of the present achievements in nano-MRI bridging the gap between the nano-MRI community and those in the medical field that will ultimately benefit from the further development of the techniques discussed. NV center magnetometry has the potential to revolutionize MRI in the longer term. The use of nanocrystals within current MRI protocols provides many short-term opportunities worth further development and application.
